# Epidemiology, clinical characteristics, and treatment outcomes of patients with COVID-19 at Thailand’s university-based referral hospital

**DOI:** 10.1186/s12879-021-06081-z

**Published:** 2021-04-26

**Authors:** Rujipas Sirijatuphat, Yupin Suputtamongkol, Nasikarn Angkasekwinai, Navin Horthongkham, Methee Chayakulkeeree, Pinyo Rattanaumpawan, Pornpan Koomanachai, Susan Assanasen, Yong Rongrungruang, Nitipatana Chierakul, Ranistha Ratanarat, Anupop Jitmuang, Walaiporn Wangchinda, Wannee Kantakamalakul

**Affiliations:** 1 Division of Infectious Diseases and Tropical Medicine, Department of Medicine, Faculty of Medicine Siriraj Hospital, Mahidol University, Bangkok, Thailand; 2 Department of Microbiology, Faculty of Medicine Siriraj Hospital, Mahidol University, Bangkok, Thailand; 3 Division of Respiratory Diseases and Tuberculosis, Department of Medicine, Faculty of Medicine Siriraj Hospital, Mahidol University, Bangkok, Thailand; 4 Division of Critical Care, Department of Medicine, Faculty of Medicine Siriraj Hospital, Mahidol University, Bangkok, Thailand

**Keywords:** COVID-19, Epidemiology, Clinical characteristics, Treatment, Outcome, Thailand

## Abstract

**Background:**

The epidemiology and outcomes of COVID-19 patients in Thailand are scarce.

**Methods:**

This retrospective cohort study included adult hospitalized patients who were diagnosed with COVID-19 at Siriraj Hospital during February 2020 to April 2020.

**Results:**

The prevalence of COVID-19 was 7.5% (107 COVID-19 patients) among 1409 patients who underwent RT-PCR for SARS-CoV-2 detection at our hospital during the outbreak period. Patients with COVID-19 presented with symptoms in 94.4%. Among the 104 patients who were treated with antiviral medications, 78 (75%) received 2-drug regimen (lopinavir/ritonavir or darunavir/ritonavir plus chloroquine or hydroxychloroquine), and 26 (25%) received a 3-drug regimen with favipiravir added to the 2-drug regimen. Disease progression was observed in 18 patients (16.8%). All patients with COVID-19 were discharged alive.

**Conclusions:**

The prevalence of COVID-19 was 7.5% among patients who underwent RT-PCR testing, and 10% among those having risk factors for COVID-19 acquisition. Combination antiviral therapies for COVID-19 patients were well-tolerated and produced a favorable outcome.

## Background

Coronavirus disease 2019 (COVID-19), which is an acute viral respiratory infection that is caused by severe acute respiratory syndrome coronavirus-2 (SAR-CoV-2), has been rapidly spreading across the world since the first patient was identified in December 2019, and it has evolved into a full-blown pandemic within a few months [1]. As of 7 June 2020, the World Health Organization (WHO) reported 7,553,182 confirmed cases of COVID-19 worldwide, with 423,349 deaths or a case fatality rate of 6%. Since COVID-19 emerged in Thailand on 13 January 2020, there have been only 3134 confirmed cases with a relatively low case fatality rate of 2% [1].

Patients with COVID-19 generally present with mild symptoms, but manifestations can vary ranging from asymptomatic to severe condition with acute respiratory distress syndrome (ARDS) [2, 3]. The mainstay treatment for COVID-19 is supportive care. Given the recent emergence of COVID-19 and the fact that established treatment guidelines have not yet been determined, many different antiviral agents (chloroquine, hydroxychloroquine, lopinavir/ritonavir, remdesivir, and favipiravir) have been recommended in several international guidelines for clinical trial purposes [3–5]. In Thailand, antiviral treatment is recommended for all symptomatic COVID-19 patients. A combination regimen of two or three antiviral medications, including protease inhibitors (lopinavir/ritonavir or darunavir/ritonavir), 4-aminoquinoline agents (chloroquine or hydroxychloroquine), and favipiravir was selected according to disease severity and the presence of risk factors for disease progression.

The epidemiology of COVID-19 might vary according to several factors, location/country, weather, national health policy, and social awareness [1–3]. There are few published studies about the epidemiology and clinical characteristics of patients with COVID-19 in Thailand, and most of those publications are case series or case reports [6–9].

The aim of this study was to determine the epidemiology, clinical characteristics, treatment and clinical outcomes of adult patients with COVID-19 at Siriraj Hospital – Thailand’s largest university-based tertiary care center.

## Methods

The medical records of all hospitalized patients aged ≥18 years who were diagnosed with COVID-19 at Siriraj Hospital during the 1 February 2020 to 30 April 2020 were reviewed. Siriraj Hospital is a 2300-bed national tertiary referral center that is located in Bangkok, Thailand.

Diagnosis of COVID-19 is made based on the detection of ≥2 SARS-CoV-2 genes by reverse transcription polymerase chain reaction (RT-PCR) from nasopharyngeal (NP) swab, throat swab, and/or any respiratory samples [10]. Briefly, after collection of the NP or throat swab, the specimen was placed into viral transport media (VTM). RNA was extracted using a magLEAD® 12gC automated extraction platform (Precision System Science, Chiba, Japan). Allplex™ 2019-nCoV Assay (Seegene, Seoul, South Korea) was used for SARS-CoV detection, which includes three gene targets (E, RdRp, and N). Briefly, 8 μL of extracted RNA was added to 5 μL of 5X Real-time One-step Buffer, 5 μL of 2019-nCoV MOM, 2 μL of Real-time One-step Enzyme, and 5 μL of RNase-Free Water. A CFX-96 real-time thermal cycler (Bio-Rad Laboratories, Inc., Hercules, CA, USA) was used for amplification. The conditions consisted of 1 cycle of 20 min at 50 °C and then 1 min at 95 °C, followed by 45 cycles of 15 s at 94 °C and 45 cycles of 30 s at 58 °C. The result was analyzed using a Seegene Viewer (Seegene).

According to the Thailand national clinical practice guidelines for treatment of COVID-19, combined regimen of antiviral medications was selected based on disease severity and the presence of risk factors for disease progression. Patients with one or more of the following are considered to be at risk for disease progression: age > 60 years or < 5 years, chronic pulmonary disease, chronic kidney disease, cardiovascular disease, cerebrovascular disease, hypertension, diabetes mellitus, obesity (body mass index [BMI] ≥35 kg/m^2^), cirrhosis, immunocompromised status, and/or lymphocyte count < 1000 cells/mm^3^, as well as the severity of illness (mild and presence of pneumonia). Briefly, two-drug antiviral treatment with a combination of a protease inhibitor (lopinavir/ritonavir or darunavir/ritonavir) and a 4-aminoquinoline agent (chloroquine or hydroxychloroquine) is considered for treatment of patients with mild disease regardless of the risk factors for disease progression. Three-drug antiviral therapy with the addition of favipiravir to the two-drug regimen is recommended for patients with COVID-19 pneumonia. Both the 2- and 3-drug combination therapies should be given for at least 5 days, but the duration can be extended to as long as 10 days based on patient’s clinical response. All patients with confirmed COVID-19 must be hospitalized for at least 14 days after symptom onset, and must be isolated for another 14 days at home or at designated facilities [11].

Data that were collected from patient medical records included demographic data, clinical features, underlying illnesses, baseline laboratory parameters, chest X-ray, antiviral therapy, oxygen support, intensive care unit (ICU) stay, and outcome of treatment. The date of disease onset was defined as the day when the first symptom was observed. Pneumonia was defined as fever and/or respiratory symptoms with appearance of new or progressive infiltrate on chest imaging.

The disease severity of COVID-19 was classified as 1) mild (defined as no pneumonia or mild pneumonia); 2) severe (defined as presence of dyspnea, respiratory rate ≥ 30/min, oxygen saturation (SpO_2_) ≤93%, and PaO_2_/FiO_2_ ≤ 300 mmHg; or, 3) critical (defined as acute respiratory failure/acute respiratory distress syndrome (ARDS), septic shock, and/or multi-organ dysfunction) [12, 13]. Disease progression was defined as new onset of severe disease or critical disease after hospitalization.

### Statistical analysis

Data are presented as number and percentage for categorical data, and as mean ± standard deviation for normally distributed data or median and range for non-normally distributed data. Fisher’s exact test or chi-square test was used to compare qualitative variables, and *t*-test or Mann-Whitney U test was used to compare quantitative variables. Variables with a *p*-value < 0.05 were further analyzed for independent association with pneumonia using multiple logistic regression. All statistical analyses were performed using IBM SPSS Statistics version 20 (SPSS, Inc., Chicago, IL, USA). A *p*-value less than 0.05 was regarded as being statistically significant.

## Results

**Fig. 1:**
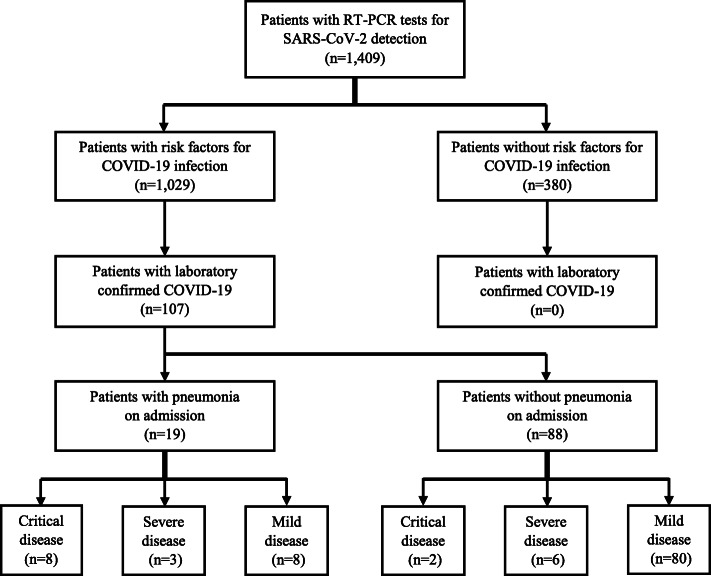
Flow diagram of patients who underwent real-time reverse transcription polymerase chain reaction (RT-PCR) test for SARS-CoV-2

Of 1409 patients who attended Siriraj Hospital and underwent RT-PCR testing for SARS-CoV-2 to evaluate for COVID-19, 1201 patients (85.2%) were symptomatic, and 208 patients (14.8%) were asymptomatic. Among those underwent RT-PCR testing, 1029 patients (73.0%) had risk factors for COVID-19 infection, including history of contact with confirmed COVID-19 patients, history of foreign travel or contact with foreigners, and/or history of visiting crowded public areas (Fig. 1). There were 107 adult patients with laboratory-confirmed COVID-19 (7.5%; 95% confidence interval [CI]: 6.2–9.0%), and 96 of those patients (89.7%) were diagnosed during 15 March 2020 to 14 April 2020.

### Patient characteristics

Among the 107 patients with COVID-19, 101 patients (94.4%) presented with symptomatic infection, and 6 patients (5.6%) presented with asymptomatic infection. The proportion of COVID-19 among symptomatic patients was 8.4% (95% CI: 6.8–10.0%), whereas the proportion of COVID-19 among asymptomatic patients was 2.8% (95% CI: 0.6–5.2%). All patients with COVID-19 in our hospital had risk factors for acquiring SARS-CoV-2 infection. Eighty-six patients (80.4%) had a history of contact with confirmed COVID-19 patients from the clusters of COVID-19 outbreak in Thailand (boxing stadiums and pubs), and their families; 15 patients (14.0%) were imported infections; and, 6 patients (5.6%) had a history of visiting crowded public areas (markets, hotels, buses, and restaurants). The proportion of COVID-19 among patients with risk factors for COVID-19 infection was 10.4% (95% CI: 8.5–12.3%).

**Fig. 2:**
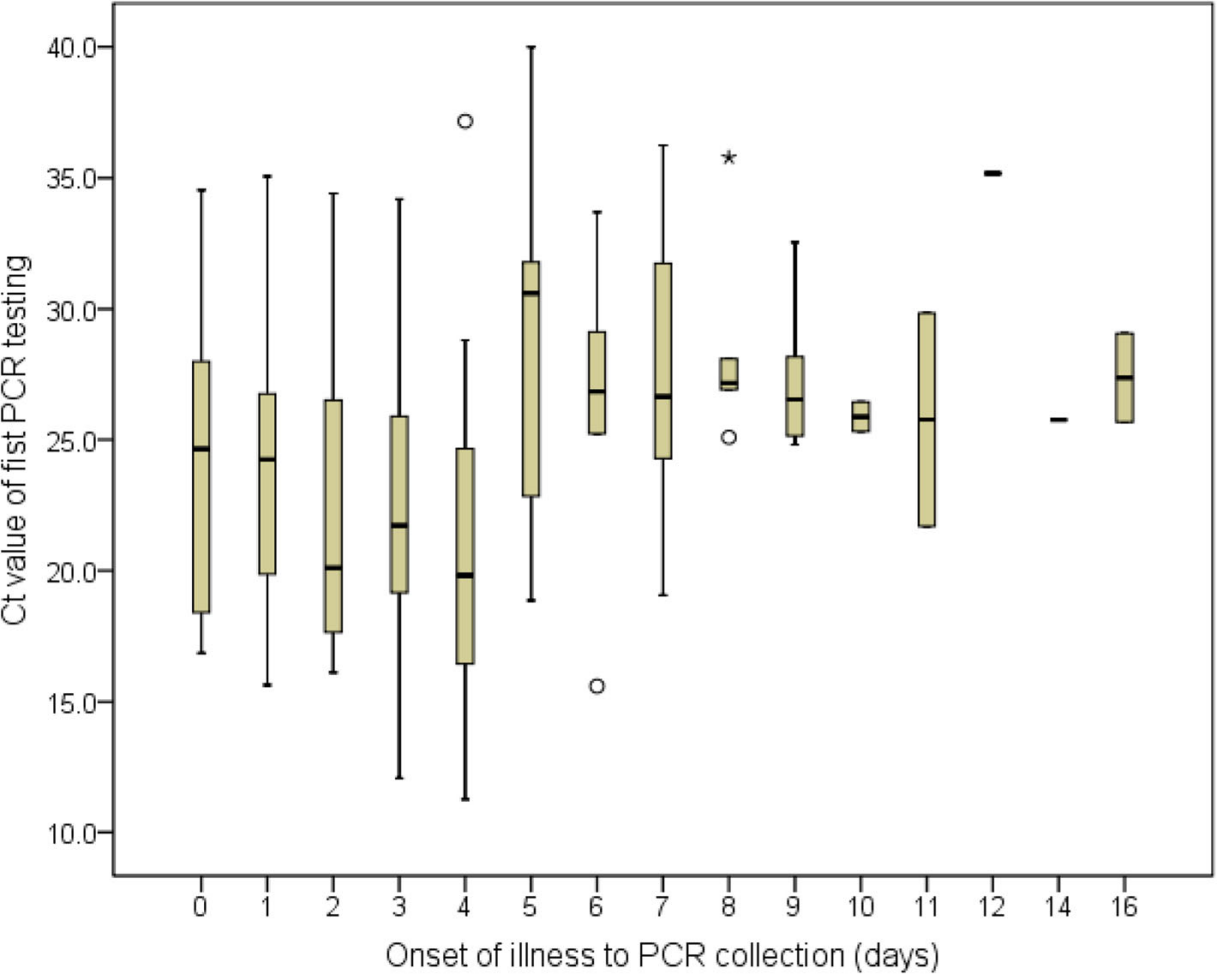
Cycle threshold (Ct) of first PCR test and onset of illness

**Fig. 3:**
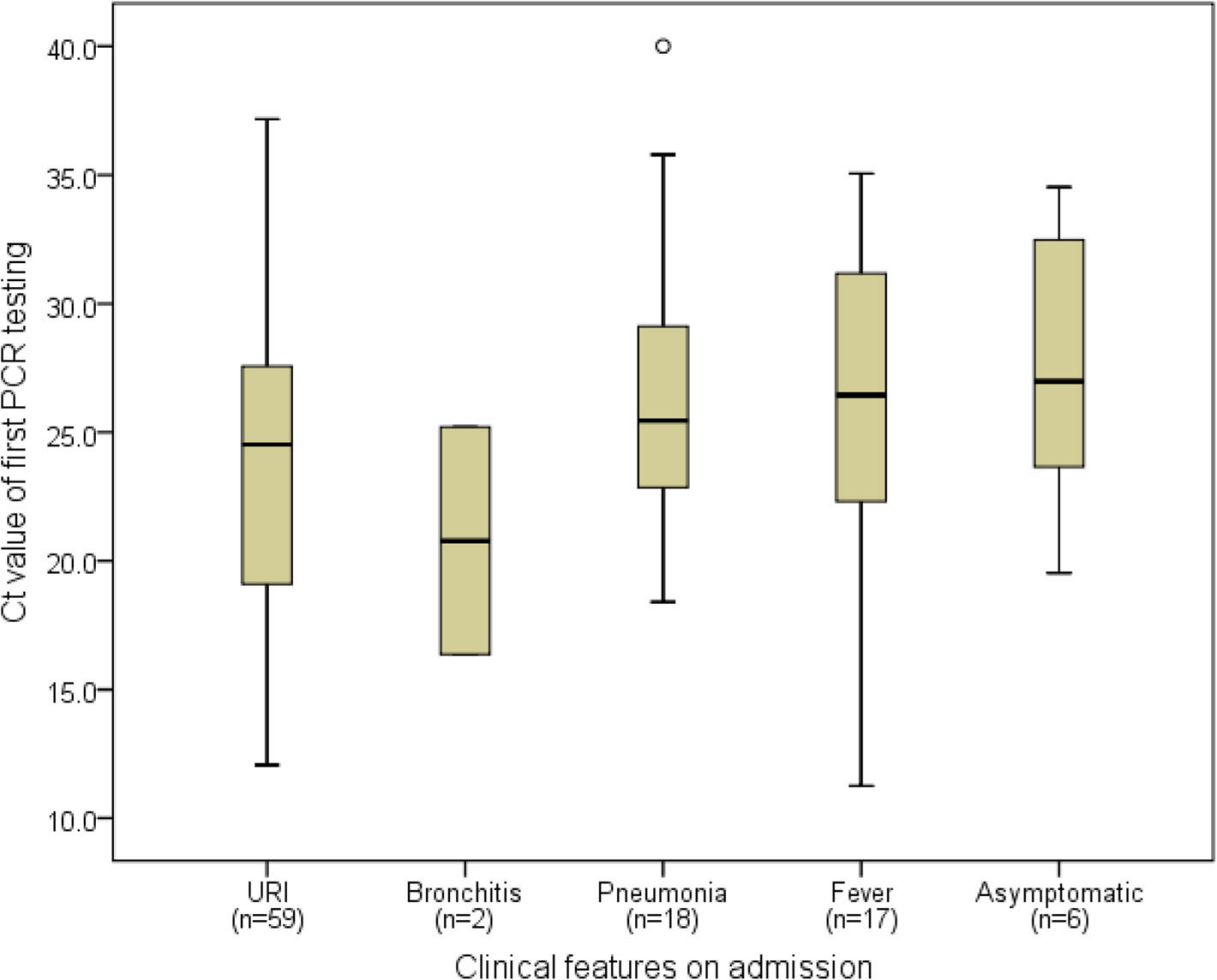
Cycle threshold (Ct) of first PCR test and clinical features on admission (URI, upper respiratory infection)

Among 102 patients with the result of their first PCR test available, the median cycle threshold (Ct) value of PCR was 25.2 (range: 11.3–40.0), and the median time from onset of illness to specimen collection was 3 days (range: 0–16). The Ct value of first PCR testing and timing from onset of illness to specimen collection are shown in Fig. 2. Sixty-two patients (60.8%) from whom a specimen was collected within less than 5 days of symptom onset had a significantly lower median Ct value than the 40% of patients who had a specimen collected at 5 or more days after symptom onset (median Ct value of 21.9 [range: 11.3–37.2] vs. 27.0 [range: 15–40]; *p* < 0.001). No significant difference in baseline Ct value (*p* = 0.192) was found when compared among different clinical features on admission (Fig. 3).

### Comparison of COVID-19 patients with and without pneumonia

The clinical characteristics, laboratory findings, treatments, and outcomes of all COVID-19 patients, and compared between those with and without pneumonia are shown in Tables 1 and 2. Of the 27 patients with COVID-19 pneumonia (25.2%), 19 were diagnosed with pneumonia at the time of admission, and 8 patients developed pneumonia during hospitalization. Median time from symptom onset to the development of pneumonia was 7 days (range: 1–18). Among the 19 patients who were diagnosed with pneumonia at the time of admission, bilateral opacities were found in 13 patients (68.4%), and the other 6 patients (31.6%) had unilateral involvement. All 8 patients who developed pneumonia thereafter had bilateral involvement on their chest X-ray. Patients with COVID-19 pneumonia were older, had a higher BMI, more likely to have comorbidity, more likely to have symptom, such as fever, cough, dyspnea and desaturation, lymphocytopenia, higher C-reactive protein (CRP), lower albumin, and higher globulin than those without pneumonia. Multivariate analysis revealed lymphocytopenia < 1000/mm^3^ (odds ratio [OR]: 21.4; 95% CI: 2.8–162.9; *p* = 0.003) and high CRP level (OR: 45.7; 95% CI: 9.4–222.2; *p* < 0.001) to be independently associated with pneumonia.

**Table 1:** Characteristics and laboratory findings of all confirmed COVID-19 patients, and compared between those with and without pneumonia

Characteristics	All patients	Patients with pneumonia	Patients without pneumonia	***p***-value^*^
	(***n*** = 107)	(***n*** = 27)	(***n*** = 80)	
	n (%)	n (%)	n (%)	
Male gender	63 (58.9%)	18 (66.7%)	45 (56.3%)	0.342
Median age, y	38 (20–83)	51 (24–83)	32.5 (20–72)	< 0.001
Age > 50 y	32 (29.9%)	14 (51.9%)	18 (22.5%)	0.004
Median BMI, kg/m^2^	23.1 (18.1–46.1)	25.0 (19.2–43.8)	22.8 (18.1–46.1)	0.007
BMI ≥25 kg/m^2^	37 (34.6%)	14 (51.9%)	23 (28.8%)	0.029
Risk factor for COVID-19 infection				
Contact confirmed COVID-19 patients	86 (80.6%)	20 (74.1%)	66 (82.5%)	0.340
Imported patients^a^	15 (14.0%)	4 (14.8%)	11 (13.8%)	0.890
Visiting public area	6 (5.6%)	3 (11.1%)	3 (3.8%)	0.151
Presence of comorbidities	31 (29.0%)	14 (51.9%)	17 (21.3%)	0.002
Diabetes mellitus	12 (11.2%)	7 (25.9%)	5 (6.3%)	0.005
Hypertension	11 (10.3%)	7 (25.9%)	4 (5.0%)	0.002
Dyslipidemia	6 (5.6%)	1 (3.7%)	5 (6.3%)	0.619
Liver disease	4 (3.7%)	0 (0.0%)	4 (5.0%)	0.183
Malignancy	4 (3.7%),	4 (14.8%)	0 (0.0%)	< 0.001
Neurologic disease	3 (2.8%)	2 (7.4%)	1 (1.3%)	0.094
Heart disease	2 (1.9%)	1 (3.7%)	1 (1.3%)	0.416
Lung disease	2 (1.9%)	0 (0.0%)	2 (2.5%)	0.407
Kidney disease	1 (0.9%)	0 (0.0%)	1 (1.3%)	0.559
Presenting symptoms				
Fever/history of fever	83 (77.6%)	26 (96.3%)	57 (71.3%)	0.007
Median BT, °C	37.2 (35.7–39.5)	37.5 (36.6–38.9)	37.2 (35.7–39.5)	0.053
BT ≥37.5 °C	48 (44.8%)	18 (66.7%)	30 (37.5%)	0.008
Cough	64 (58.9%)	20 (74.1%)	44 (55.0%)	0.080
Sore throat	39 (36.4%)	9 (33.3%)	30 (37.5%)	0.697
Myalgia	28 (26.2%)	5 (18.5%)	23 (28.8%)	0.296
Rhinorrhea	24 (22.4%)	4 (14.8%)	20 (25.0%)	0.273
Dyspnea	23 (21.5%)	15 (55.6%)	8 (10.0%)	< 0.001
Headache	16 (15.0%)	2 (7.4%)	14 (17.5%)	0.204
Productive sputum	12 (11.2%)	4 (14.8%)	8 (10.0%)	0.493
Diarrhea	10 (9.3%)	4 (14.8%)	6 (7.5%)	0.259
Desaturation^b^	6 (5.6%)	6 (22.2%)	0 (0.0%)	< 0.001
Clinical features at the time of admission				
URI	61 (57.0%)	3 (11.1%)	58 (72.5%)	< 0.001
Pneumonia	19 (17.8%)	19 (70.4%)	0 (0.0%)	< 0.001
Acute febrile illness	18 (16.8%)	2 (7.4%)	16 (20.0%)	0.130
Bronchitis	2 (1.9%)	2 (7.4%)	0 (0.0%)	0.014
Acute diarrhea	1 (0.9%)	0 (0.0%)	1 (1.3%)	0.559
Asymptomatic infection	6 (5.6%)	1 (3.7%)	5 (6.3%)	0.619
Incubation period, d	5 (1–19)	6.5 (1–15)	4 (1–19)	0.307
Time from symptom onset to admission, d	4 (0–18)	5 (1–18)	4 (0–15)	0.166
Scoring of severity on the day of admission				
NEWS2 score ≥ 5	10 (9.3%)	9 (33.3%)	1 (1.3%)	< 0.001
CRB-65 score ≥ 2	5 (4.7%)	5 (18.5%)	0 (0.0%)	< 0.001
qSOFA score ≥ 2	8 (7.5%)	7 (25.9%)	1 (1.3%)	< 0.001
Baseline laboratory findings				
Hemoglobin, g/dL	13.9 (9.5–17.4)	14.0 (9.9–17.4)	13.7 (9.5–17.4)	0.581
WBC count, /mm^3^	5070	5190	5035	0.331
	(2040-13,180)	(3380-13,180)	(2040-11,310)	
Lymphocyte count, /mm^3^	1559 (80–3231)	1108 (80–2569)	1752 (490–3231)	< 0.001
< 1000/mm^3^	14 (13.1%)	11 (40.7%)	3 (3.8%)	< 0.001
Atypical lymphocytosis	17 (15.9%)	8 (29.6%)	9 (11.3%)	0.024
Platelet count, /mm^3^	217,000	191,000	221,000	0.166
	(104,000-562,000)	(137,000-562,000)	(104,000-555,000)	
C-reactive protein, mg/L	6.2 (0.6–350.0)	35.9 (0.6–350.0)	2.8 (0.6–41.8)	< 0.001
CRP > 10 mg/L	49 (45.8%)	24 (88.9%)	13 (16.3%)	< 0.001
Total bilirubin, mg/dL	0.6 (0.1–2.3)	0.6 (0.1–1.3)	0.5 (0.1–2.3)	0.211
Direct bilirubin, mg/dL	0.2 (0.1–0.8)	0.3 (0.1–0.7)	0.2 (0.1–0.8)	0.076
AST, U/L	23 (9–284)	31 (16–79)	21 (9–284)	0.002
ALT, U/L	24 (7–162)	26 (10–140)	24 (7–162)	0.277
Alkaline phosphatase, U/L	64 (27–121)	66 (32–121)	64 (27–102)	0.287
Albumin, g/dL	4.3 (2.6–5.0)	4.0 (2.6–5.0)	4.3 (3.6–5.0)	< 0.001
Globulin, g/dL	3.2 (2.3–4.5)	3.6 (2.7–4.5)	3.1 (2.3–4.3)	< 0.001
Blood urea nitrogen, mg/dL	11.6 (4.0–40.3)	12.2 (4.0–40.3)	11.5 (5.4–29.2)	0.427
Creatinine, mg/dL	0.8 (0.4–2.5)	0.8 (0.4–1.6)	0.8 (0.5–2.5)	0.250

Continuous data presented as median and range

A *p*-value< 0.05 indicates statistical significance

^*^Comparison between patients with and without COVID-19 pneumonia

^a^Patients with history of foreign travel or contact with foreigner

^b^Desaturation defined as SpO_2_ ≤ 93%

**Abbreviations:** *BMI* body mass index; *BT* body temperature; *WBC* white blood cell count; *AST* aspartate aminotransferase; *ALT* alanine aminotransferase; *NEWS2* National Early Warning Score 2; CRB-65, Confusion, Respiratory rate ≥ 30/min, systolic blood pressure < 90 mmHg or diastolic blood pressure ≤ 60 mmHg, age ≥ 65 years; *qSOFA* quick Sepsis-related Organ Failure Assessment; *URI* upper respiratory infection

**Table 2:** Treatments and outcomes of all confirmed COVID-19 patients, and compared between those with and without pneumonia

Treatments / Outcomes	All patients	Patients with pneumonia	Patients without Pneumonia	***p***-value^*^
	(***n*** = 107)	(n = 27)	(n = 80)	
	n (%)	n (%)	n (%)	
***Treatments***				
Receiving 2-drug antiviral therapy^a^	78 (75.0%)	3 (11.1%)	75 (93.8%)	< 0.001
Receiving 3-drug antiviral therapy^b^	26 (25.0%)	24 (88.9%)	2 (2.5%)	< 0.001
No antiviral treatment	3 (2.8%)	0 (0%)	3 (3.8%)	0.307
Any antibiotic treatment^c^	13 (12.1%)	11 (40.7%)	2 (2.5%)	< 0.001
Empiric antibiotic therapy	10 (9.3%)	9 (33.3%0	1 (1.3%)	< 0.001
Azithromycin treatment	8 (7.5%)	4 (14.8%)	4 (5.0%)	0.094
***Outcomes of hospitalization***				
Diarrhea after hospitalization	32 (29.9%)	7 (25.9%)	25 (31.3%)	0.601
Nausea after hospitalization	24 (22.4%)	5 (18.5%)	19 (23.8%)	0.573
ICU stay	13 (12.1%)	13 (48.1%)	0 (0.0%)	< 0.001
Median (range) duration of ICU stay, d	9 (4–38)	9 (4–38)	N/A	NA
Receiving oxygen support	23 (21.5%)	21 (77.8%)	2 (2.5%)	< 0.001
Oxygen cannula	10 (9.3%)	8 (29.6%)	2 (2.5%)	< 0.001
High-flow oxygen cannula	6 (5.6%)	6 (22.2%)	0 (0.0%)	< 0.001
Non-invasive mechanical ventilation	2 (1.9%)	2 (7.4%)	0 (0.0%)	0.010
Invasive mechanical ventilation	5 (4.7%)	5 (18.5%)	0 (0.0%)	< 0.001
Median duration of ET, d	11 (5–17)	11 (5–17)	N/A	N/A
Median (range) length of stay, d	11 (2–38)	14 (6–38)	10.5 (2–35)	< 0.001
Clinical outcome				
Discharged alive	107 (100%)	27 (100%)	80 (100%)	NA

A *p*-value < 0.05 indicates statistical significance

^*^Comparison between patients with and without COVID-19 pneumonia

^a^2-drug antiviral therapy: lopinavir/ritonavir or darunavir/ritonavir plus chloroquine or hydroxychloroquine

^b^3-drug antiviral therapy: lopinavir/ritonavir or darunavir/ritonavir plus chloroquine or hydroxychloroquine plus favipiravir

^c^Some patients received more than 1 course of antibiotic treatment

**Abbreviations:** *ICU* intensive care unit; *ET* endotracheal tube; *NA* not applicable

### Treatment and outcome

Among the 104 patients (97.2%) treated with antiviral medications, 78 (75.0%) received 2-drug antiviral therapy. Of those, 32 patients (41.0%) received lopinavir/ritonavir plus chloroquine, and 46 patients (59.0%) received darunavir/ritonavir plus hydroxychloroquine. Twenty-six patients (25.0%) received 3-drug antiviral therapy due to presence of pneumonia. The 3-drug regimen consisted of the 2-drug regimen plus favipiravir. Three patients (2 with URI and 1 with asymptomatic infection) received only symptomatic treatment. During treatment, 32 patients (29.9%) developed diarrhea with a median duration of 2 days after treatment (range: 1–3), and 24 patients (22.4%) developed nausea. However, all of those patients were clinically improved after symptomatic treatment.

Respiratory virus panels and bacterial cultures from respiratory samples were sent in 20 and 13 patients with community-acquired pneumonia, respectively; however, no respiratory coinfection was identified. No patients with COVID-19 received concurrent oseltamivir therapy. Antibiotic treatment was given in only 13 patients with COVID-19 (12.1%), and 3 patients received > 1 course of antibiotic treatment. Ten patients received empiric antibiotic therapy for community-acquired infections (azithromycin in 4 patients, ceftriaxone plus azithromycin in 4 patients, and levofloxacin in 2 patients). Six patients received antibiotic treatment for nosocomial infections. Among those 6 patients, 5 received antibiotics for treatment of nosocomial pneumonia (meropenem in 3 patients, and piperacillin/tazobactam in 2 patients), and one patient received cefdinir and metronidazole for treatment of perianal abscess. One out of six patients who received antibiotic developed antibiotic-associated diarrhea, and was treated with oral vancomycin.

At the time of hospital discharge, 88 patients (82.2%) were classified as mild disease (80 non-pneumonia, and 8 mild pneumonia). Nine patients (8.4%) were classified as severe disease due to oxygen desaturation (SpO_2_ ≤ 93%) (1 patient had severe disease, and 8 patients had mild disease at the time of admission). The other 10 (9.3%) were classified as critical disease due to the development of ARDS (5 patients had severe disease, and 5 patients had mild disease at the time of admission).

Disease progression during hospitalization was observed in 18 patients (16.8%; 8 patients with severe disease, and 10 patients with critical disease). Median time from symptom onset to disease progression was 11 days (range: 4–16). Compared to those without disease progression, patients with disease progression were older (52.5 vs. 33 years, *p* < 0.001), had a higher median BMI (26.3 vs. 22.9 kg/m^2^, *p* < 0.01), presence of underlying disease (66.7 vs. 21.3%, *p* < 0.001), pneumonia since first admission (55.6 vs. 10.1%, *p* < 0.001), lower baseline lymphocyte count (1071 vs. 1690/mm^3^, *p* < 0.001), higher baseline CRP (61.2 vs. 3.8 mg/L, *p* < 0.001), lower albumin (3.8 vs. 4.3 g/dL, *p* < 0.001), and higher globulin (3.8 vs. 3.1 g/dL, *p* < 0.001).

Several interventions were administrated as adjunctive therapy in 12 COVID-19 patients who had severe or critical disease, including corticosteroid in 5 patients, corticosteroid with tocilizumab in 2 patients, corticosteroid with hemoperfusion in 2 patients, tocilizumab in 2 patients, and hemoperfusion in 1 patient. Multivariate analysis revealed presence of underlying disease (OR: 6.7, 95% CI: 1.7–26.2; *p* = 0.006) and high CRP level (OR: 55.2, 95% CI: 6.7–465.2; *p* < 0.001) to be independent factors associated with disease progression. All patients with COVID-19 in our hospital were clinically improved and discharged alive. The median length of stay was 11 days (range: 2–38), and was significantly longer among those with pneumonia (14 vs. 10.5 days, *p* < 0.001).

## Discussion

COVID-19 continues to cause a global health and economic crisis due to its uncontrolled spreading and the lack of an approved vaccine and treatment option. However, the COVID-19 situation in Thailand remains comparatively stable with a relatively lower fatality rate than many other counties. A combination of two or three antiviral drugs is recommended for all symptomatic COVID-19 patients in Thailand.

The present study revealed the following important findings. First, the prevalence of COVID-19 at our hospital was approximately 10% among patients with risk factors for COVID-19 acquisition during the COVID-19 outbreak in Bangkok, Thailand. No COVID-19 patient was documented among those without risk factors for COVID-19 acquisition. This finding is different from the epidemiologic reports from other countries where the outbreak situation of SARS-CoV-2 is ongoing [14]. The very low prevalence of COVID-19 among patients without risk factors for disease acquisition is also good supporting evidence for effective control of disease outbreak in Thailand. It should be noted that healthcare facilities in Thailand take an approach of contact tracing, which is a critical step in stopping COVID-19 transmission. After COVID-19 cases are confirmed, healthcare personnel collaborate with public health officials to identify all persons who have been in close proximity with the case, and those people are monitored daily for 14 days. This may explain why we always identified at least one risk factor for COVID-19 acquisition in COVID-19 patients.

Second, the major presenting symptoms in our patients with COVID-19 were fever or history of fever and cough. In contrast, we found rhinorrhea or sore throat to be uncommon, which is similar to many reports from other countries [12, 13, 15]. In addition, almost 17% of COVID-19 patients in our study presented with acute febrile illness without any respiratory symptoms, which is similar to the findings from China [15]. This may create significant concerns of misdiagnosis when co-epidemics of COVID-19 and other tropical diseases, such as dengue, occur due to the similarity of clinical and laboratory findings, such as leukopenia. Third, the majority of our patients presented with mild disease as evidenced by the fact that less than 10% of all patients had a baseline National Early Warning Score 2 (NEWS2) ≥5 or quick Sepsis-related Organ Failure Assessment (qSOFA) ≥2. However, 18% of patients still had clinical progression to severe or critical disease during hospitalization, which is similar to the findings of a previous large study conducted in China [12, 13, 15].

The next important point is that the mortality rate was 0% in this study. This may be explained by several reasons. First, our patients were younger and had milder disease compared with patients included in other studies [12, 15]. This can, in part, be explained by the fact that many confirmed cases in our study were linked to two major clusters of COVID-19 outbreak in Bangkok, Thailand, boxing stadiums and pubs. Approximately 6% of our patients were also asymptomatic at the time of admission. In addition, our patients were all immediately admitted to the hospital upon detection of SAR-CoV-2 for clinical observation and management since it’s mandatory in Thailand to admit all COVID-19 patients to prevent transmission to other individuals.

In addition, we found COVID-19 pneumonia to be significantly associated with lymphocytopenia and a high CRP level. This finding is similar to that from a previous meta-analysis [16]. In addition, subsequent disease progression was found in approximately 17% of patients in this study, and the presence of underlying disease and high CRP level were identified as independent factors associated with disease progression. Lastly, most of the patients with COVID-19 at our hospital received antiviral treatment, including protease inhibitor and antimalarial drugs with or without favipiravir, and both the 2- and 3-drug regimens were quite well-tolerated with no reported serious adverse events. Even though the risk of QT prolongation from hydroxychloroquine was increased in previous study (particularly when being used concurrently with azithromycin), we did not find any serious cardiac events in our patients, which may be due to younger age, milder disease, and less comorbidity compared with previous study [17].

The strength of this study is that our research shows the clinical and laboratory data from a cohort of patients that acquired COVID-19 during the outbreak period in Thailand. Moreover, the favorable tolerability and clinical outcome of using recommended 2- or 3-drug antiviral regimens for all symptomatic COVID-19 patients according to the Thai national clinical practice guideline (CPG) was demonstrated in this study. The limitation of our study is that the medical record reviewer was not blinded, which could have introduced some unintended bias. Furthermore, we were unable to collect some data, such as EKG results, procalcitonin level, and lactate dehydrogenase (LDH) level, in all patients since these parameters were not routinely tested in patients with COVID-19. Finally, this study was conducted at a single institution, so our results may not be generalizable to other hospitals or care settings.

## Conclusions

The prevalence of COVID-19 was 10% among patients with risk factors for COVID-19 acquisition at our university hospital during the outbreak period. Most COVID-19 patients had mild disease, and approximately 18% had severe or critical disease. A combination regimen of two or three antiviral drugs was administered in all symptomatic COVID-19 patients, and all prescribed regimens were well-tolerated and yielded a favorable outcome.

## Data Availability

The datasets used and/or analysed during the current study are available from the corresponding author on reasonable request.

## Abbreviations

COVID-19: Coronavirus disease 2019
SAR-CoV-2: Severe acute respiratory syndrome
WHO: World health organization
ARDS: Acute respiratory distress syndrome
RT-PCR: Reverse transcription polymerase chain reaction
NP: Nasopharyngeal swab
VTM: Viral transport media
BMI: Body mass index
ICU: Intensive care unit
SpO_2_: Oxygen saturation
CI: Confidence interval
Ct: Cycle threshold
CRP: C-reactive protein
NEWS2: National early warning score 2
qSOFA: quick sepsis-related organ failure assessment
CPG: Clinical practice guideline
LDH: Lactate dehydrogenase
